# Patch-based adaptive weighting with segmentation and scale (PAWSS) for visual tracking in surgical video

**DOI:** 10.1016/j.media.2019.07.002

**Published:** 2019-10

**Authors:** Xiaofei Du, Maximilian Allan, Sebastian Bodenstedt, Lena Maier-Hein, Stefanie Speidel, Alessio Dore, Danail Stoyanov

**Affiliations:** a Wellcome / EPSRC Centre for Interventional and Surgical Sciences (WEISS), University College London, UK; b Intuitive Surgical Inc., USA; c Karlsruhe Institute of Technology, Karlsruhe, Germany; d Division of Computer-Assisted Medical Interventions (CAMI), German Cancer Research Center (DKFZ), Heidelberg, Germany; e Deliveroo, London, UK

**Keywords:** Visual object tracking, Tracking-by-detection, Computer assisted interventions, Surgical instrument tracking

## Abstract

Vision-based tracking in an important component for building computer assisted interventions in minimally invasive surgery as it facilitates estimation of motion for instruments and anatomical targets. Tracking-by-detection algorithms are widely used for visual tracking, where the problem is treated as a classification task and a tracking target appearance model is updated over time using online learning. In challenging conditions, like surgical scenes, where tracking targets deform and vary in scale, the update step is prone to include background information in model appearance or to lack the ability to estimate change of scale, which degrades the performance of classifier. In this paper, we propose a Patch-based Adaptive Weighting with Segmentation and Scale (PAWSS) tracking framework that tackles both scale and background problems. A simple but effective colour-based segmentation model is used to suppress background information and multi-scale samples are extracted to enrich the training pool, which allows the tracker to handle both incremental and abrupt scale variations between frames. Experimentally, we evaluate our approach on Online Tracking Benchmark (OTB) dataset and Visual Object Tracking (VOT) challenge datasets, showing that our approach outperforms recent state-of-the-art trackers, and it especially improves successful rate score on OTB dataset, while on VOT datasets, PAWSS ranks among the top trackers while operating at real-time frame rates. Focusing on the application of PAWSS to surgical scenes, we evaluate on MICCAI 2015 challenge instrument tracking challenge and in vivo datasets, showing that our approach performs the best among all submitted methods and also has promising performance on in vivo surgical instrument tracking.

## Highlights

• A simple but effective colour-based segmentation model is incorporated to assign weight to the patch-based descriptor.
• A two-level sampling strategy enables the tracker to handle both incremental and abrupt scale variations.
• Achieve superior results on various datasets among top trackers with near real-time performance.
• Substantial evaluation is performed on both ex-vivo and in-vivo surgical datasets.

## Graphical abstract

**Figure:**
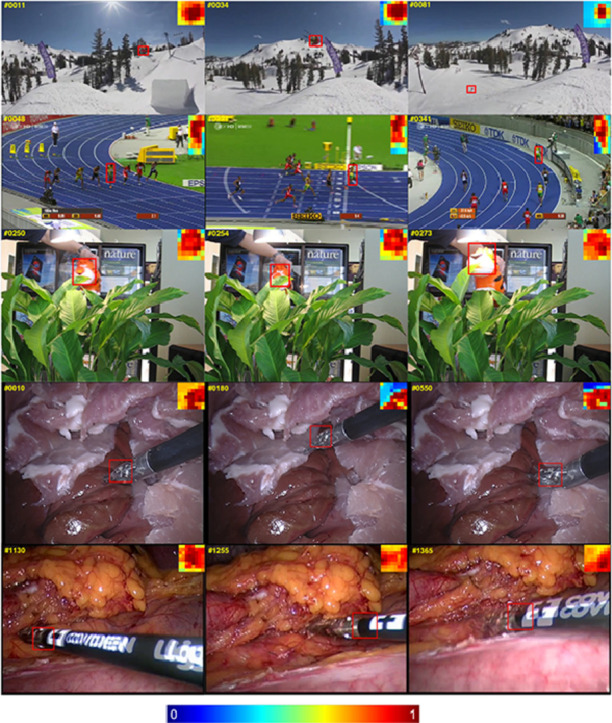

## 1 Introduction

**Fig. 1:**
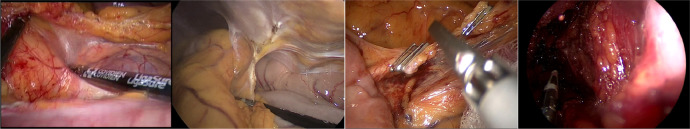
Challenges of object tracking in surgical scenes, including image blur, tissue occlusion, dramatic scale and lighting variations.

Minimally invasive surgery (MIS) relies on endoscopic and laparoscopic video cameras to provide the surgeon with vision inside the body. Developing computer assistance for such procedures with multi-modal image overlays, robotics or novel imaging requires tracking of a variety of structures within the surgical site to estimate their motion and update their position. Visual tracking in an appealing approach for this task because it relies only on the existing camera and it provides information within the surgeon’s reference view. But visual tracking in surgical scenes involves significant challenges, especially for long term targets. Several frame samples are displayed in Fig. 1. Take the surgical instrument as a tracking target, it may disappear from the scene or be occluded by tissue via manipulation, also its appearance may significantly changes due to image blurring, bleeding, lighting and scale variations.

The key components of a successful tracking algorithm includes the target representation and how to update the representation over time. In this paper, we incorporate a Patch-based Adaptive Weighting with Segmentation and Scale (PAWSS) into tracking-by-detection, resulting a pragmatic framework, focusing on simple but effective algorithms. Given the initial position (bounding box) of a target, PAWSS divides the target into non-overlapping patches. By using a simple but effective colour-based segmentation model, each patch is assigned with a weight which decreases background information influences within the bounding box. Besides, a two-level sampling strategy is introduced to extract multi-scale samples, which enables the tracker to handle both incremental and abrupt scale variations between frames. To reference our method to general tracking approaches, we evaluated and compared it with state-of-the-art methods on Online Tracking Benchmark (OTB) (Wu et al., 2013) and VOT challenge datasets. To show how it performs for surgical scenes, we used MICCAI 2015 instrument tracking datasets with promising results demonstrating that PAWSS is the best performing tracker, which also works in real-time without any specific code optimisation.

## 2 Related work

**Tracking-by-detection:** Recently, inspired by the success of object detection algorithms, tracking-by-detection methods has been taking inspiration from advances in machine learning, such as structured output support vector machines (SVM) (Tsochantaridis et al., 2005), boosting (Avidan, 2007, Grabner, Grabner, Bischof, 2006), Gaussian process regression (Gao et al., 2014) and deep learning (Wang et al., 2015). Tracking-by-detection frameworks build a classifier to distinguish the tracked object from background and update this classifier with new positive observations as well as with negative information. It is inevitable that falsely labelled samples will appear and degrade the model because wrongly labelled samples of background confuse the classifier ultimately leading to drift or failure. Structured Output Tracking with Kernels (Struck) (Hare et al., 2011) adopts a structured output SVM and circumvents the traditional collection of positive and negative samples by integrating the labelling procedure within the learning process. In recent benchmark (Wu et al., 2013) Struck has shown excellent tracking performance compared to prior work.

**Patch-based Representations:** Recently patch-wise descriptors have been exploited to represent the object appearance (Kim, Lee, Sim, Kim, 2015, Chen, Yuan, Wu, Zhang, Zheng, 2013, Zhang, van der Maaten, 2014). A bounding box is divided into cells or patches and low-level features are used to construct features of these patches, which represent local structural information. A major challenge for tracking-by-detection methods is that the bounding box usually not only includes the object but also some background information. Background changes differently to the moving object and causes inaccurate information transfer through the model update. To address this problem, different methods have been proposed to decrease the effects of background information such as assigning different weights based on the pixel spatial location or appearance similarity (Comaniciu, Ramesh, Meer, 2003, He, Yang, Lau, Wang, Yang, 2013, Lee, Sim, Kim, 2014). SOWP (Kim et al., 2015) exploits this concept by incorporating Random Walk with Restart (RWR) simulations to assign weights to patches. RWR simulations exploit the similarity between neighbouring patches and their relevance or self-similarity to the object appearance. Stationary distributions can be obtained to represent likelihoods that each patch belongs to either foreground or background. Patch weights are designed according to likelihoods so that foreground patches would have relatively larger weights. We introduce a different weighting method to patches by incorporating a colour-based segmentation model. Previous papers have integrated a segmentation step into tracking (Godec, Roth, Bischof, 2013, Duffner, Garcia, 2013), but these methods are sensitive to segmentation results since they directly track the segmented object patches free from the constraints of bounding box. By applying a segmentation step to patch weights instead we manage to enhance performance and avoid this sensitivity.

**Surgical instrument tracking:** For surgical instrument tracking, information from different sources has been used for instrument tracking. Typically colour, gradient or texture (Uecker, Wang, Lee, Wang, 1995, Cano, Gayá, Lamata, Sánchez-González, Gómez, 2008) is employed to represent the appearance model. The work (Reiter and Allen, 2010) proposed to learn the instrument appearance online by combining multiple features, and explores new areas as the instrument moves in or out of view. To make feature of the instrument more distinctive, artificial markers were designed and mounted to the instrument (Wei, Arbter, Hirzinger, 1997, Zhang, Payandeh, 2002, Tonet, Thoranaghatte, Megali, Dario, 2007, Zhang, Ye, Chan, Yang, 2017). Although attaching markers on instrument makes tracking more robust and simple, the idea of modifying instruments is usually avoided since it changes the surgical procedure. Also, artificial markers may introduce inconvenience, such as biological hazard or retrofittable difficulty. Instrument shape can be simplified or explored using a prior model to confine the search space (Pezzementi et al., 2009). To classify the target from background, a random forest was learnt to classify instrument in pixel-wise fashion, then the binary classification output was used to estimate the pose of a prior 3D instrument model through optimization within a level set framework (Allan et al., 2013). Then, it was improved by combining constraints from feature points, temporal motion model with stereo setup (Allan et al., 2014). Multi-part appearance model (Allan et al., 2015) and articulated degrees-of-freedom (Allan et al., 2018) of robotic instruments can be used to align the prior model with low level optical flow constraints. In addition, cues such as robotic kinematics (Ye et al., 2016) can also be used as external constraints.

## 3 Proposed algorithm

### 3.1 Patch-based descriptor

**Fig. 2:**
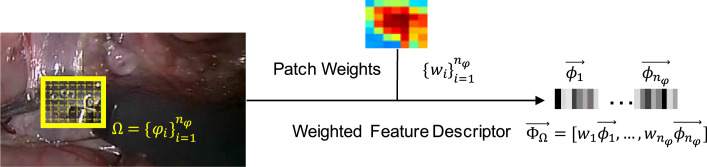
Patch-based descriptor ${\vec{\Phi }}_{\Omega }$. Given a bounding box Ω, it is equally decomposed into *n*_φ_ patches ${\left\{\varphi_{i}\right\}}_{i=1}^{n_{\varphi }}$. For the i-th patch φ_*i*_, low-level feature vector *ϕ_i_* is extracted, and is assigned with a weight *w_i_*. Then, the descriptor ${\vec{\Phi }}_{\Omega }$ is constructed by concatenating features of all patches, weighted by patch weights. Note that example patch weights are shown by the highlighted bounding box. Warmer colour indicates higher weight value.

Given the location (bounding box Ω) of the object, to represent the object appearance, we used patch-based descriptor shown in Fig. 2. Ω is evenly decomposed into *n*_φ_ non-overlapping patches ${\left\{\varphi_{i}\right\}}_{i=1}^{n_{\varphi }}$. Low-level feature vector $\vec{\phi }$ is extracted for each patch. Patch-based descriptor of Ω can be constructed by concatenating features of all the patches in their spatial order. Since background information is potentially included in the bounding box, we incorporate an global probabilistic segmentation model (Collins, Liu, Leordeanu, 2005, Duffner, Garcia, 2013) to assign weights ${\left\{w_{i}\right\}}_{i=1}^{n_{\varphi }}$ to the patches based on their colour appearance, resulting a weighted descriptor:

(1) $${\vec{\Phi }}_{\Omega }=\left[w_{1}{\vec{\phi }}_{1},\cdots ,w_{n_{\varphi }}{\vec{\phi }}_{n_{\varphi }}\right]$$

where *w_i_* is the weight of the feature ${\vec{\phi }}_{i}$ of the *i*-th patch φ_*i*_.

### 3.2 Probabilistic segmentation model for patch weighting

The global segmentation model is based on colour histogram by using a recursive Bayesian formulation to discriminate foreground and background. Let *y*_1: *t*_ be the colour observation of a pixel from frame 1 to *t, c* be the class of a pixel. In our application, a pixel is classified as foreground (c=1) or background (c=0) by its colour observation. The foreground probability distribution $p(c_{t}=1|y_{1:t})$ at frame *t* is based on tracked results from previous frames

(2) $$\begin{array}{ccc} & & p\left(c_{t}=1|y_{1:t}\right)=Z^{-1}p\left(y_{t}|c_{t}=1\right)\\ & & \;\sum_{c_{t-1}}p\left(c_{t}=1|c_{t-1}\right)p\left(c_{t-1}|y_{1:t-1}\right)\\ & & \;p\left(c_{t}=1|c_{t-1}=1\right)=0.6\;p\left(c_{t}=1|c_{t-1}=0\right)=0.4 \end{array}$$

where *c_t_* is the class of a pixel at frame *t*: 0 for background, and 1 for foreground, and *Z* is a normalization constant, which can be ignored in practice. The transition probabilities for foreground and background $p(c_{t}|c_{t-1})$ where *c* ∈ {0, 1} are empirical choices as in Duffner and Garcia (2013). Foreground histogram $p(y_{t}|c_{t}=1)$ and background histogram $p(y_{t}|c_{t}=0)$ are initialized from all the pixels inside the bounding box and from those which are surrounding the bounding box (with some margin between) in the first frame, respectively. For the following frames, the colour histogram distributions are updated using tracked result.

(3) $$p\left(y_{t}|c_{t}=1\right)=\delta p\left(y_{t}|y_{t}\in \Omega_{t}\right))+\left(1-\delta \right)p\left(y_{t-1}|c_{t-1}=1\right)$$

where 0 ≤ *δ* ≤ 1 is the model update factor. Ω_*t*_ represents tracked bounding box in frame *t*. Instead of treating every pixel equal, the weighting of a pixel also depends on the patch where it is located. Patches with higher weight are more likely to contain object pixels and vice versa. So the colour histogram update for colour observation *y_t_* of current frame *t* is defined as

(4) $$p\left(y_{t}|y_{t}\in \Omega_{t}\right)=\frac{\sum_{i=1}^{n_{\varphi }}w_{i,t-1}N_{y_{t}\in \varphi_{i,t}}}{\sum_{i=1}^{n_{\varphi }}w_{i,t-1}\sum_{x_{t}}N_{x_{t}\in \varphi_{i,t}}}$$

where $N_{y_{t}\in \varphi_{i,t}}$ represents the number of pixels with colour observation *y_t_* in the *i*-th patch φ_*i,t*_ in frame *t*, and *x_t_* represents any colour observation in frame *t*, so the denominator means the weighted number of all the pixel colour observations in the bounding box Ω_*t*_.

The weights *w*_*i*, 1_ for all the patches are initialized as 1 at the first frame, and then are updated based on the segmentation model

(5) $$w_{i,t}=\delta {\bar{w}}_{i,t}+\left(1-\delta \right)w_{i,t-1}$$

(6) $${\bar{w}}_{i,t}=\frac{\varpi_{i,t}}{\max_{1\leq i\leq n_{\varphi }}\varpi_{i,t}}$$

(7) $$\varpi_{i,t}=\frac{\sum_{x_{t}}p\left(x_{t}|c_{t}=1\right)N_{x_{t}\in \varphi_{i,t}}}{\sum_{x_{t}}N_{x_{t}\in \varphi_{i,t}}}$$

where ϖ_*i,t*_ denotes the average foreground probability of all pixels in the patch φ_*i,t*_ in the current frame *t*, it is normalized so the highest weight update ${\bar{w}}_{i,t}$ equals 1. The patch weight *w*_*i, t*_ is then updated gradually over time. We omit background probability distribution $p(c_{t}=0|y_{1:t})$ since it is similar to Eq. (2).

**Fig. 3:**
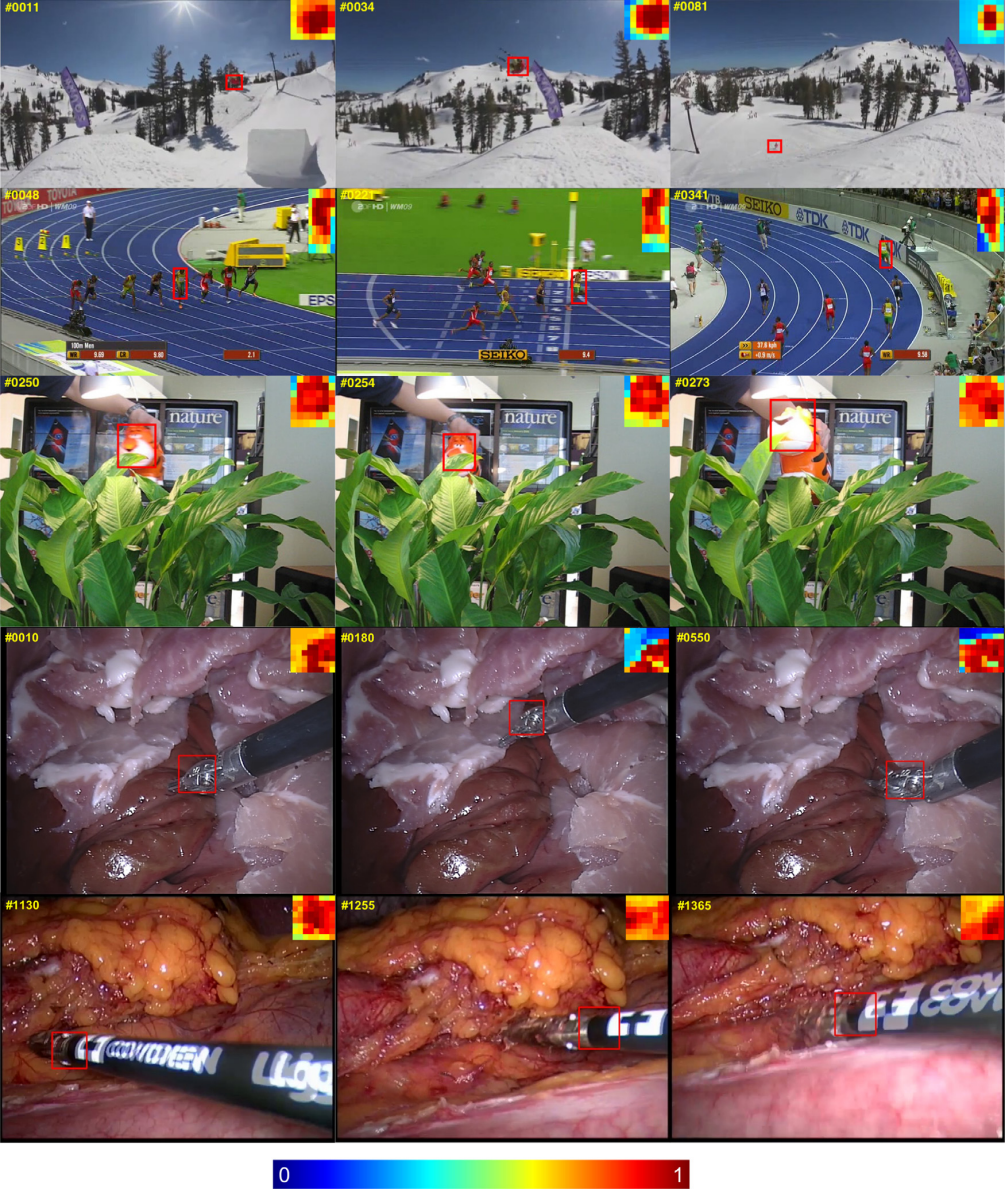
Example patch weights are shown for the highlighted bounding box displayed in the top corner of the image. Warmer colour indicates higher foreground possibility.

Unlike the weighting strategy in other patch-based methods (Chen, Yuan, Wu, Zhang, Zheng, 2013, Kim, Lee, Sim, Kim, 2015) by analysing the similarities between neighbouring patches, our patch weighting method is simple and straightforward to implement, the weight update for each patch is independent from each other, and only relies on the colour histogram based segmentation model. We show examples of the patch weight development in Fig. 3. The patch weight thumbnails are displayed on the top corner of each frame, which indicate the objectness in the bounding box and also reflect the object deformation over time. Since we update the segmentation model based on previous patch weights, and in turn the segmentation model facilitates updating the weight of all patches. This co-training strategy enhances the weight contrast between foreground and occluded patches, which suppresses background information efficiently.

### 3.3 Two-level sampling for scale estimation

The tracked object often undergoes complicated transformations during tracking, for example, deformation, scale variations, occlusion, etc. Fixed-scale bounding box estimation is ill-equipped to capture the accurate extents of the object, which would degrade the classifier performance by providing samples which are either partial cropped or include background information.

**Fig. 4:**
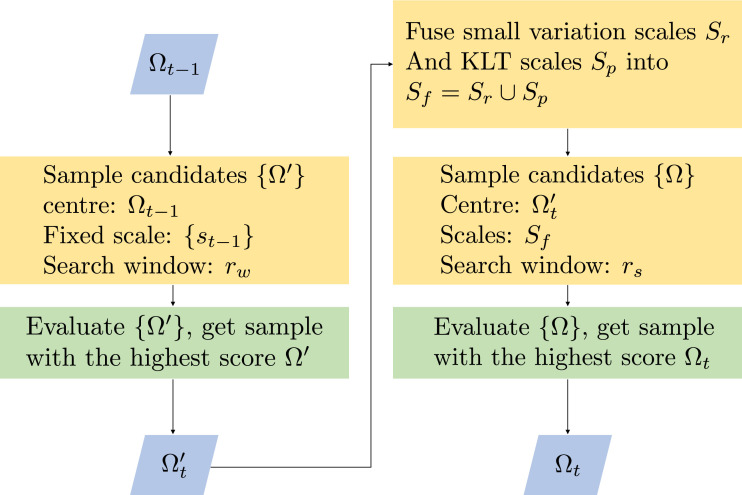
Two-level sampling strategy workflow.

When locating the object in a new frame, all the bounding box candidates are collected within a search window, and the bounding box with the maximum classification score is selected to update the object location. Rather than making a suboptimal decision by choosing from fixed-scale samples, we augment training sample pool with multi-scale candidates, which is referred as two-level sampling strategy (see Fig. 4). On the first level, all the bounding box samples are extracted with fixed-scale $s_{t-1}$ (the object scale in frame t−1). The search window is centered at the $\Omega_{t-1}$ with a height/width of *r_w_*, then the weighted patch-based descriptor of all candidates {Ω′} are fed into the classifier, and we select the bounding box $\Omega_{t}^{\prime }$ with the maximum classification score not as the final decision, but as the search center for our second level. After first level, the rough location of the object is narrowed to a smaller area. We then set a smaller search window with search height/width of *r_s_*, centring at the bounding box $\Omega_{t}^{\prime }$ selected in the first level, and we construct multi-scale candidates {Ω} within the search window. All the samples are evaluated by the classifier, and we select the bounding box Ω_*t*_ of the sample with the maximum score as the final location of the object.

Obviously, the scales of augmented samples are critical. We consider two complementary strategies that handle both incremental and abrupt scale variations. Firstly, to deal with relatively small scale changes between frames, we build a scale set *S_r_*

(8) $$S_{r}=\left\{s|s=\lambda^{m}s_{t-1}\right\}\;m\in \left[-\frac{n_{r}-1}{2},\cdots ,\frac{n_{r}-1}{2}\right]$$

where *λ* is a fixed value which is slightly larger than 1.0. It is set to accurately search the scale change. *n_r_* is the scale number in the scale set *S_r_*. $s_{t-1}$ is the scale of the object in frame t−1 compared with the initial bounding box in the first frame. Considering object scale usually does not vary too much between frames, scale set *S_r_* includes scales which are close to the previous frame.

Secondly, when object undergoes abrupt scale changes between frames, scale set *S_r_* is unable to keep pace with the speed of the scale variations. To address this problem, we build an additional scale set *S_p_* by incorporating Lucas–Kanade tracker (KLT) (Bouguet, 2001, Shi, Tomasi, 1994), which helps us estimate the scale change explicitly. We randomly pick *n_pt_* points from each patch in the bounding box $\Omega_{t-1}$ of frame t−1, and tracked all these points in the next frame *t*. With sufficient well-tracked points, we can estimate the scale variation between frames by comparing the distance changes of the tracked point pairs.

**Fig. 5:**
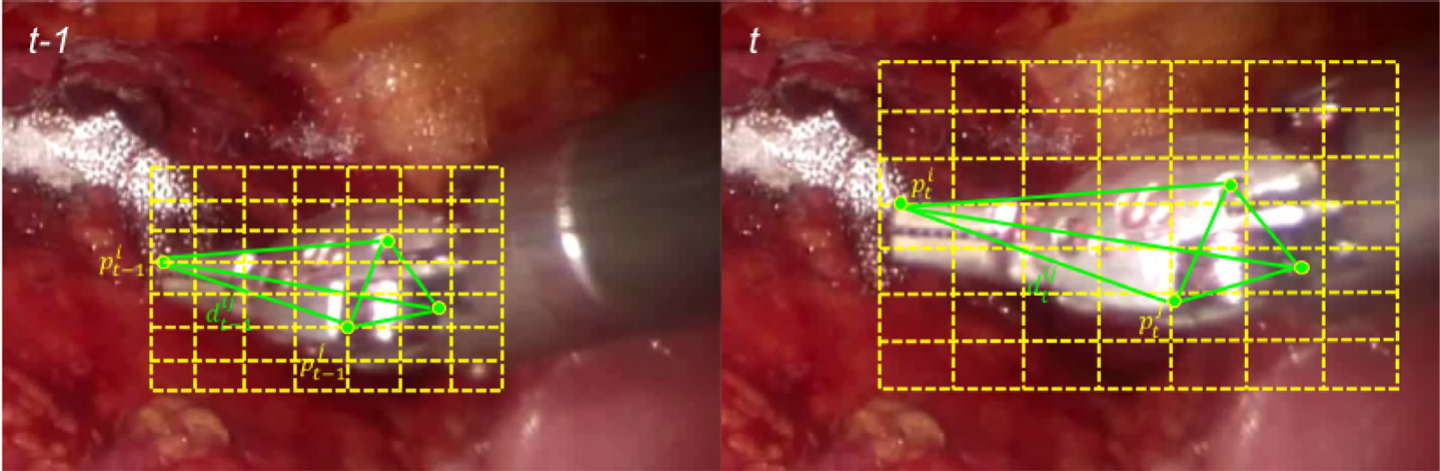
Illustration of scale estimation by using the KLT tracker. Random points located on the patches are picked in frame t−1, and are tracked in the next frame *t* by the KLT tracker, the distance ratio of point pairs (*p^i^, p^j^*) between two frames are used for scale estimation. We use 7 × 7 patch grids, resulting *n*_φ_=49 in the illustration.

We illustrated the scale estimation by KLT tracker in Fig. 5. Let $p_{t-1}^{i}$ denotes one picked point in the previous frame t−1 and its matched point $p_{t}^{i}$ in the current frame *t*. We compute the distance $d_{t-1}^{ij}$ between point-pair $(p_{t-1}^{i},p_{t-1}^{j}),$ and the distance $d_{t}^{ij}$ between the matched point-pair $\left(p_{t}^{i},p_{t}^{j}\right)$. For all the matched point pairs, we compute the distance ratio between the two frames

(9) $$V=\{s|s=d_{t}^{ij}/d_{t-1}^{ij}\}\;i\neq j$$

where *V* is the set with all the distance ratios. We sort *V* by value and pick the median element $s_{p}=V_{sorted}\left(\frac{n}{2}\right)$ as the potential scale change of the object. To make scale estimation more robust, we uniformly sample the scales ranging between [1, *s_p_*] or [*s_p_*, 1] to construct the scale set *S_p_*.

(10) $$S_{p}=\left\{s|s=1+i\frac{s_{p}-1}{n_{p}-1}\right\}\;0\leq i<n_{p}$$

where *n_p_* is the scale number in the scale set *S_p_*. When the object is out-of-view, occluded or abruptly deforms, the ratio of well-tracked points will be low. In that case, the estimation from the KLT tracker will be unreliable. In our implementation, when the ratio is lower than 0.5, we then set $s_{p}=1,$ therefore the scale set *S_p_* will only add samples with the previous scale into the candidate pool. Only when there are enough points well tracked, the estimation from the KLT tracker will be trusted. We fuse these two complementary scale sets *S_r_* and *S_p_* into $S_{f}=S_{r}\cup S_{p}$ to enrich our sample candidate pool. To show the effectiveness, we evaluate our proposed tracker in Section 4 with or without scale set *S_p_* estimated by the KLT tracker.

### 3.4 Tracking framework

**Fig. 6:**
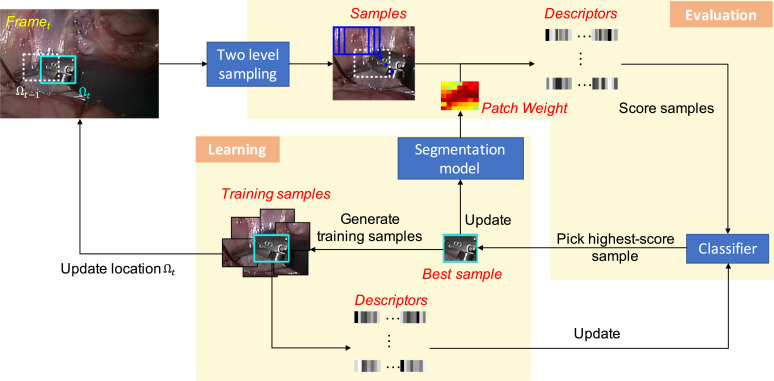
Tracking framework. Given the target location $\Omega_{t-1}$ in the previous frame at time t−1, the framework is to predict the target location Ω_*t*_ in the current frame at time *t*. The framework includes evaluation and learning phases. In evaluation phase, multi-scale samples are extracted via two-level sampling strategy, and then are fed into the classifier to pick the one with the highest score. The location of the sample is considered as the new location Ω_*t*_. The sample is also used for updating the segmentation model and the classifier in the learning phase.

PAWSS can be combined with any tracking-by-detection method. We show the pipeline of the whole framework in Fig. 6. It includes two phases: *evaluation* and *learning*. The evaluation phase is to find the target in a new frame. Given the bounding box $\Omega_{t-1}$ in the previous frame t−1, sample candidates are extracted in a search window, which centers at $\Omega_{t-1}$ in the current frame *t*, unlike other tracking-by-detection approaches, we adapt a two-level sampling strategy for accurate scale estimation (Section 3.3). Via the colour-based segmentation model, weights of all patches are updated as in Section 3.2, and the descriptors of all samples are computed via patch weighting. Descriptors of all samples are fed into classifier and the one with the highest output score is picked as the best sample. The location Ω_*t*_ of the best sample shows where the target is in the current frame at time *t*. Between frames, the target appearance changes due to deformation, occlusions, light and scale variations, therefore, the classifier and the segmentation model needs to be learnt online to keep up with the changes. The best sample among all samples represents the most similar one compared to the target. For one thing, pixel colour distribution of the best sample is used to update the segmentation model. For another, samples are extracted around the best sample in order to collect foreground and background information. Descriptors of all samples are computed and used to train the classifier online to better discriminate the target from neighbouring background. The procedure starts again for the next frame.

In our implementation, we incorporate PAWSS into Struck Hare et al. (2011). The algorithm relies on an online structured output SVM learning framework which integrates learning and tracking. It directly predicts the location displacement between frame, avoiding the heuristic intermediate step for assigning binary labels to training samples, which achieves top performance in OTB dataset Wu et al. (2013).

## 4 Results

**Implementation details:** Our algorithm is publicly available online^1^ and is implemented in C++ and performs at about 7 frames per second with an i7-2.5 GHz CPU without any optimisation. We listed the parameter setting in Table 1. To illustrate the generalization of our proposed framework, we use the same parameter setting through all experiments. For structured output SVM, we are using a linear kernel and the parameters are empirically set as δ=0.1 in Eqs. (3) and (5), λ=1.003 in Eq. (8), the scale numbers of the scale set are $n_{r}=n_{p}=11$. The number of extracted points from each patch $n_{pt}=5$. The updating threshold for the classifier is set as η=0.3. For each sequence, we scale a frame to make sure the minimum side length of the bounding box is larger than 32 pixels, and the search window *r_w_* is fixed to (W+H)/2, where *W* and *H* represents the width and height of the scaled bounding box, respectively, and the search window *r_s_* is fixed to 5 pixels. We tested different low-level feature combinations in Section 4.1 and found that the combination of HSV colour and gradient features (HSV+G) achieves the best results. The patch number affects the tracking performance, too many patches increase the computation and too less patches do not robustly reflect the local appearance of the object. We tested different patch numbers, and selected $n_{\varphi }=49$ to strike a performance balance.

**Table 1:** Parameter setting of the framework in all experiments.

Number of patches *n*_φ_	7×7=49
Base of scale estimation *λ*	1.003
Number of scales for small scale changes *n_r_*	11
Number of scales for abrupt scale changes *n_p_*	11
Updating factor of classifier *η*	0.3
Updating factor of segmentation model *δ*	0.1

### 4.1 Online Tracking Benchmark (OTB)

OTB dataset (Wu et al., 2013) includes 50 sequences tagged with 11 attributes, which represent the challenging aspects for tracking such as illumination variation, occlusion, deformation et al. The tracking performance is quantitatively evaluated using both precision rate (PR) and success rate (SR), as defined in (Wu et al., 2013). PR/SR scores are depicted using precision plot and success plot, respectively. The precision plot shows the percentage of frames whose tracked centre is within certain Euclidean distance (20 pixels) from the centre of the ground truth. Success plot computes the percentage of frames whose intersection over union overlap with the ground truth annotation is within a threshold varying between 0 and 1, and the area under curve (AUC) is used for SR score. To evaluate the effectiveness of incorporating the scale set proposed by the KLT tracker, we provide two versions of our tracker as PAWSSa and PAWSSb: PAWSSa only includes scale set *S_r_*, while PAWSSb includes both *S_r_* and *S_p_* for scale estimation.

**Comparison using different features:**   Selecting right features to describe the object appearance plays a critical role in tracking. The most desirable feature property is its uniqueness so that the object can be distinguished from background. Raw intensities or colour features are usually used for histogram-based appearance representations, while edge or gradient information are less sensitive to illumination changes. Generally, many tracking approaches use a combination of these diverse features to represent the object (Hare, Saffari, Torr, 2011, Grabner, Grabner, Bischof, 2006, Li, Shen, Dick, Hengel, 2013). To evaluate the performance of our proposed approach, we tested different low-level features such as HSV colour, RGB colour, the combination of colour and gradient features (HSV+G, RGB+G) for constructing the descriptor in Table 5.1. The RGB histogram is 24-dimensional with 8 bins for each channel, and the HSV colour histogram is 20-dimensional including 8 bins for H and S channels respectively and 4 separate bins for V channel. The gradient histogram is 16-dimensional signed gradients ranging from 0 to 360^∘^. We also compared our tracker PAWSSa and PAWSSb with Struck (Hare et al., 2011) and SOWP (Kim et al., 2015). From Table 2, we observe: Augmenting colour with gradient histogram improves the tracking performance by providing diverse structural information of the object. In our experiments, the descriptor comprising combination of HSV colour and gradient features achieves the best results, we use this setting in the following evaluation.

**Table 2:** The performance of the proposed algorithm compared with different low-level features. PAWSSa and PAWSSb tracker represents our tracker without and with the KLT tracker, respectively.

	PAWSSa	PAWSSb
HSV	0.731 / 0.528	0.742 / 0.545
RGB	0.764 / 0.552	0.749 / 0.544
RGB+G	0.838 / 0.605	0.840 / 0.607
HSV+G	**0.889 / 0.635**	**0.897 / 0.649**

**Fig. 7:**
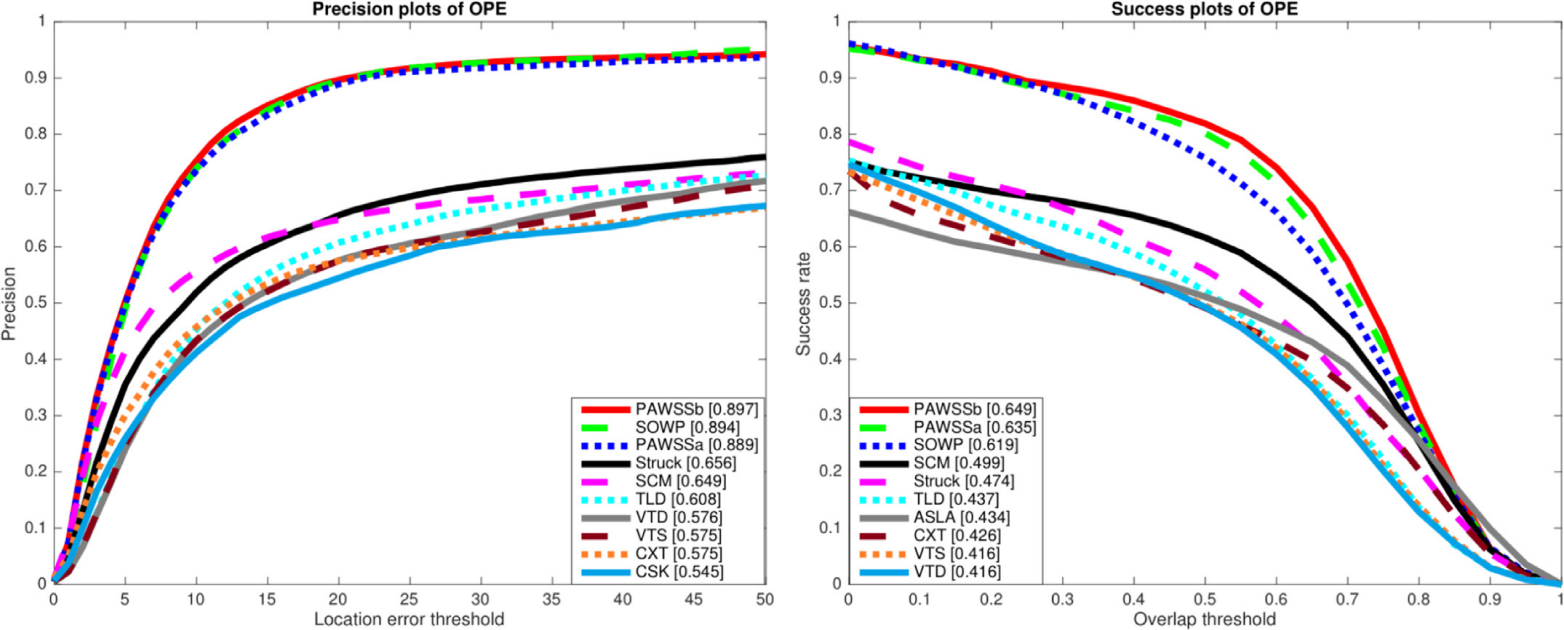
Comparison of precision and success plots on OTB with the top 10 trackers; PR scores are illustrated with the threshold at 20 pixels and SR scores with the average overlap (AUC) in the legend.

**Comparison with state-of-the-art trackers:** We use the evaluation toolkit provided by Wu et al. (2013) to generate the precision and success plots for the one pass evaluation (OPE) of the top 10 algorithms in Fig. 7. The toolkit includes 29 benchmark trackers, besides that we also include SOWP tracker. It is shown that PAWSSb achieves the best PR/SR scores among all the trackers. For a more detailed evaluation, we also compared our tracker with state-of-the-art trackers in Table 3. Notice that in all the attribute field, our tracker achieves either the best or the second best PR/SR scores. Our tracker achieves 36.7% gain in PR and 36.9% gain in SR over Struck (Hare et al., 2011). By using a simple patch weighting strategy and training with adaptive scale samples, the performance shows that our tracker provides comparable PR scores, and higher SR score compared with SOWP (Kim et al., 2015). PAWSSa tracker improves SR score by 2.6% considering gradually small changes between frames, PAWSSb improves SR score by 4.8% by incorporating scales estimated by the external KLT tracker. Specifically, when the object undergoes scare variation PAWSS achieves a performance gain of 10.3% in SR over SOWP.

**Table 3:** Comparison of PR/SR score with state-of-the-art trackers including Struck (Hare et al., 2011), DSST (Danelljan et al., 2014), SAMF (Li and Zhu, 2014), FCNT (Wang et al., 2015) and SOWP (Kim et al., 2015) in the OPE based on the 11 sequence attributes: illumination variation (IV), scale variation (SV), occlusion (OCC), deformation (DEF), motion blur (MB), fast motion (FM), in-plane rotation (IPR), out-of-plane rotation (OPR), out-of-view (OV), background cluttered (BC) and low resolution (LR). The best and the second best results are shown in **red** and **blue** colours respectively.

**Fig. 8:**
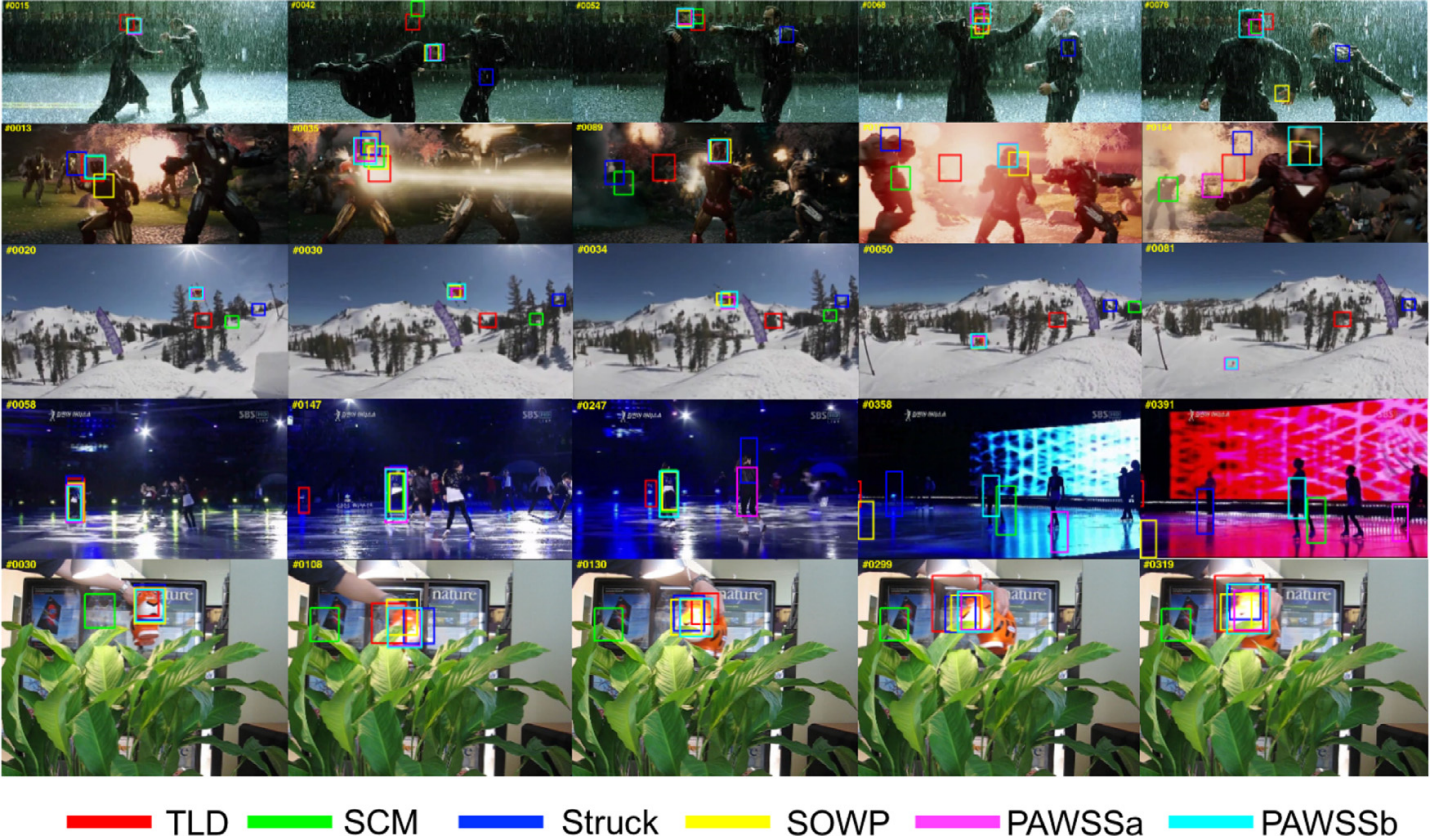
Comparison of the tracking results of our proposed tracker PAWSS with SOWP (Kim et al., 2015) and three conventional trackers: TLD (Kalal et al., 2012), SCM (Zhong et al., 2012) and Struck (Hare et al., 2011) on some especially challenging sequences in the benchmark.

**Fig. 9:**
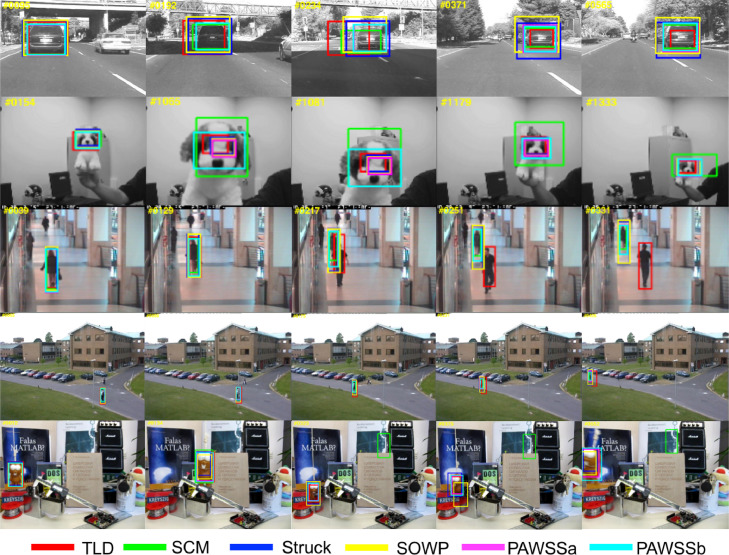
Comparison of the tracking results of our proposed tracker PAWSS with SOWP (Kim et al., 2015) and three conventional trackers: TLD (Kalal et al., 2012), SCM (Zhong et al., 2012) and Struck (Hare et al., 2011) on some sequences with scale variations in the benchmark.

We show tracking results in Figs. 8 and 9 with the top trackers including TLD (Kalal et al., 2012), SCM (Zhong et al., 2012), Struck (Hare et al., 2011), SOWP (Kim et al., 2015) and the proposed PAWSSa and PAWSSb. In Fig. 8, five challenging sequences are selected from the benchmark dataset, which include illumination variation, scale variations, deformation, occlusion or background clusters. PAWSS can adapt when the object deforms in a complicated scene and track the target accurately. In Fig. 9, we select five representative sequences with different scale variations. PAWSS can well track the object with scale variation, while other trackers drift away. The results show that our proposed tracking framework PAWSS can track the object robustly through sequence by using the weighting strategy to suppress background information within the bounding box, and also by incorporating scale estimation allowing the classifier to train with adaptive scale samples. Please see the supplementary video for more sequence tracking results.

### 4.2 Visual Object Tracking (VOT) challenges

For completeness, we also validated our algorithm on VOT2014 (25 sequences) and VOT2015 (60 sequences) datasets. VOT datasets use ranking-based evaluation methodology: Accuracy and robustness. Similar to SR rate for OTB dataset, the accuracy measures overlap of the predicted result and the ground truth bounding box, while the robustness measures how many times the tracker fails during tracking. A failure is indicated whenever the tracker loses the target object which means the overlap becomes zero, and it will be re-initialized afterwards. All the trackers are evaluated, compared and ranked based on with respect to each measure separately using the official evaluation toolkit from the challenge.^2^

**Fig. 10:**
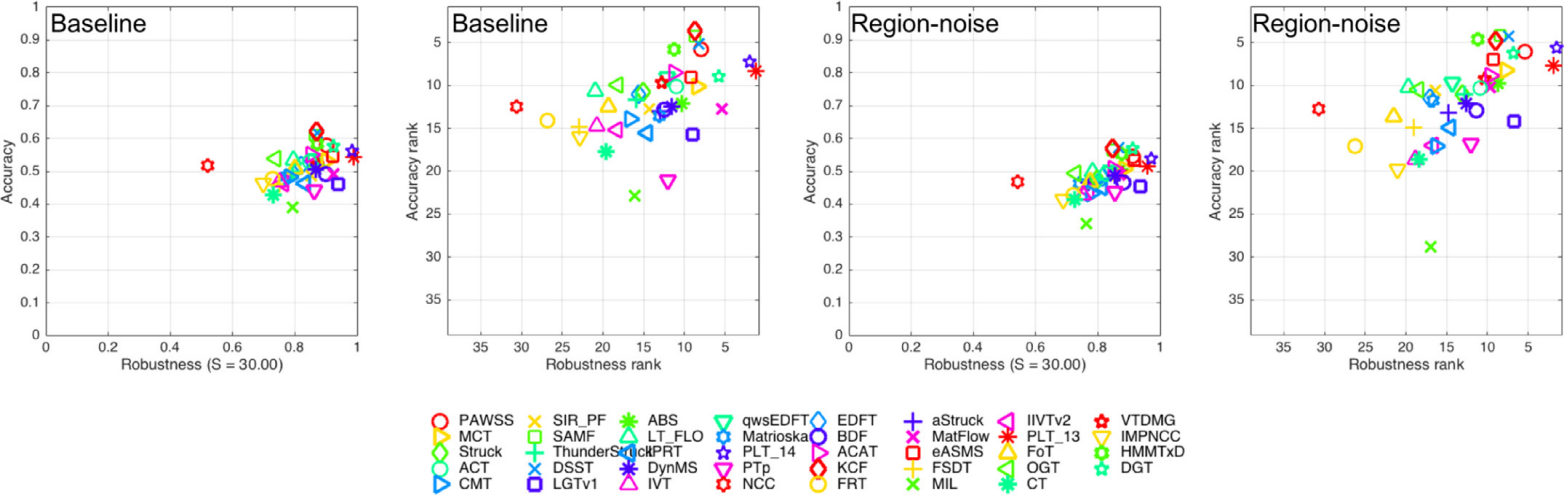
The accuracy-robustness score and ranking plots with respect to the baseline and region-noise experiments of VOT2014 dataset. Tracker is better if its result is closer to the top-right corner of the plot.

**VOT2014** VOT2014 challenge includes two experiments: Baseline experiment and region-noise experiment. In baseline experiment, a tracker runs on all the sequences by initializing with the ground truth bounding box on the first frame; while in the region-noise experiment, the tracker is initialized with a random noisy bounding box with the perturbation in the 10% of the ground truth bounding box size. (Kristan et al., 2015b). The ranking plots with 38 trackers are shown in Fig. 10 for comparing PAWSS with the top three trackers: DSST (Danelljan et al., 2014), SAMF (Li and Zhu, 2014), KCF (Henriques et al., 2015) in Table 4. For both the experiments our PAWSS has lower accuracy score 0.58/0.55, but less failures  0.88/0.78 and have a second average rank. But considering the tracking process of the experiments: once a failure is detected, the tracker will be re-initialized, to eliminate the effect of achieving higher accuracy score by more re-initialization steps, we performed experiments without the re-initialization, also shown in Table 4. The results show that PAWSS has the highest accuracy score 0.51/0.48 among all the trackers without re-initialization, which means it is more robust than the other trackers.

**Table 4:** The Accuracy (Acc.) and Robustness (Rob.) results of VOT2014 baseline and region-noise experiments with and without-re-initialization compared with the top trackers DSST (Danelljan et al., 2014), SAMF (Li and Zhu, 2014) and KCF (Henriques et al., 2015). The best and the second best results are shown in **red** and **blue** colours respectively.

**Fig. 11:**
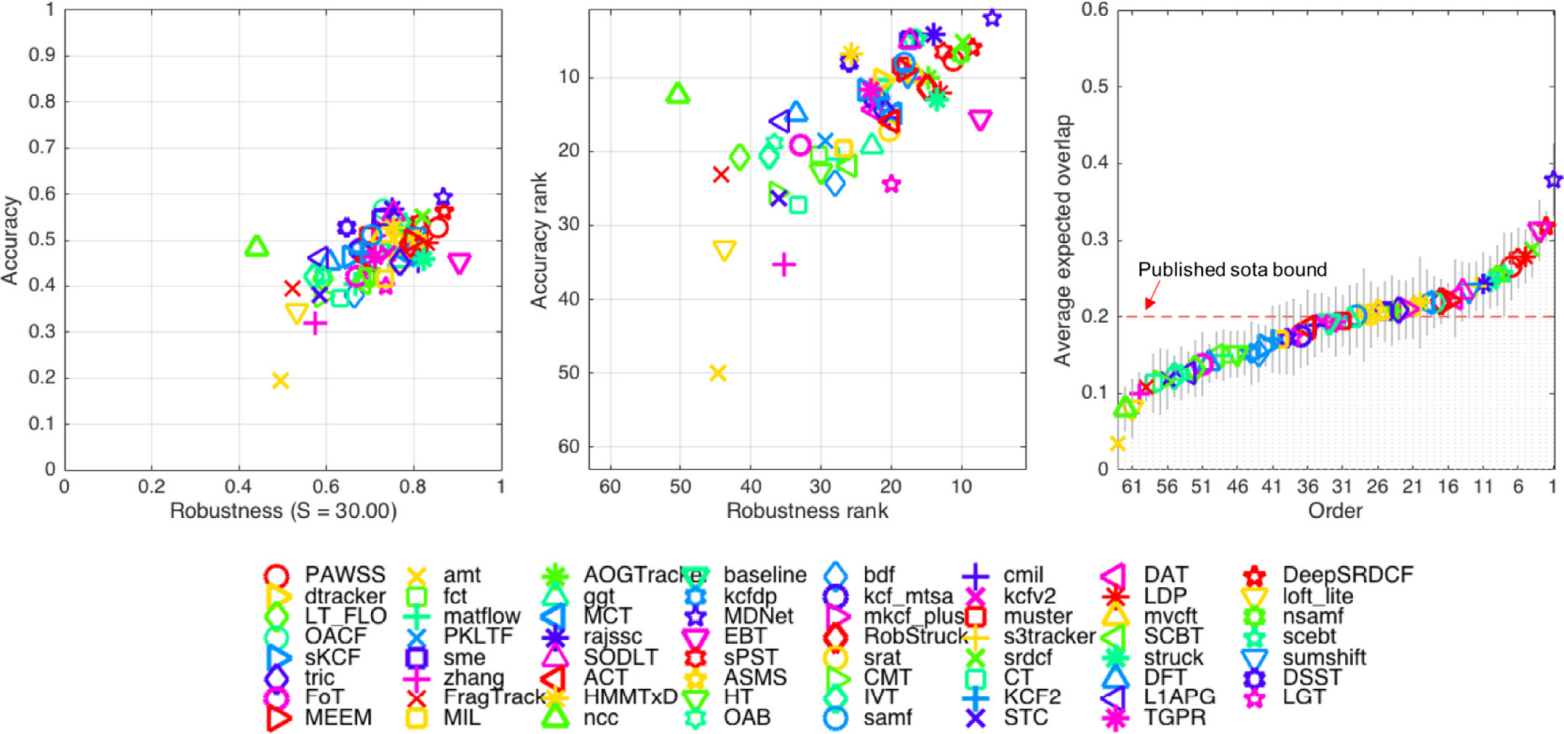
The accuracy-robustness ranking plots and the expected overlap score ranking plot of VOT2015 dataset. Tracker is better if its result is closer to the top-right corner of the plot. The published sota bound is established based on top trackers in recent years. Any tracker with performance over the boundary is considered as a state-of-the-art tracker.

**VOT2015** Finally, we evaluated and compared PAWSS with 62 trackers on VOT2015 dataset. VOT2015 challenge only includes baseline experiment, and the ranking plots are shown in Fig. 11. In VOT2013 and VOT2014, average ranking measure is used to determine the performance of the trackers. Although average ranking has taken both accuracy and robustness measure into consideration, it is not theoretically representative as a concrete tracking performance. In VOT2015 (Kristan et al., 2015a), expected average overlap measure is introduced which combines both per-frame accuracies and failures in a principled manner. Compared with the average rank, expected overlap has a more clear practical interpretation.

We list the score / rank and expected overlap of the top trackers from VOT2015 (Kristan et al., 2015a) which are either quite robust or accurate, the above VOT2014 top three trackers DSST (Danelljan et al., 2014), SAMF (Li and Zhu, 2014), KCF (Henriques et al., 2015),^3^ and the baseline NCC tracker in Table 5 and also shown in the expected average overlap plot Fig. 11. It can be shown that the average rank is not always consistent with the expected overlap. According to the paper (Kristan et al., 2015a), a VOT2015 *published sota bound* criteria (0.2) is established by averaging the tracker performance published in 2014/2015 from top computer vision conferences and journals. The tracker will be considered as a state-of-the-art tracker with performance over this boundary criteria. Our tracker PAWSS is well above the criteria, and is among those top trackers (ranks the 7-th, outperforming 54 trackers), also PAWSS achieves better than any of VOT2014 top trackers on VOT2015 dataset.

**Table 5:** VOT2015 Accuracy (Acc.), Robustness (Rob.), Score/Ranking and expected overlap results from the top trackers of VOT2014, VOT2015 and the baseline tracker. The NCC tracker is VOT2015 baseline tracker. Trackers marked with † are submitted to VOT2015 without publication.

	Baseline				Avg rank	Exp overlap
	Acc.		Rob.			
	Score	Rank	Failure	Rank		
MDNet Nam and Han (2015)	0.59	2.03	0.77	5.68	3.86	0.378
DeepSRDCF Danelljan et al. (2015a)	0.56	5.92	1.00	8.38	7.15	0.318
EBT Wang and Yeung (2014)	0.45	15.48	0.81	7.23	11.36	0.313
SRDCT Danelljan et al. (2015b)	0.55	5.25	1.18	9.83	7.54	0.288
LDP Lukežič et al. (2016)	0.49	12.08	1.30	13.07	12.58	0.279
sPST Hua et al. (2015)	0.54	6.57	1.42	12.57	9.57	0.277
**PAWSSb**	**0.53**	**7.75**	**1.28**	**11.22**	**9.49**	**0.266**
NSAMF†	0.53	7.02	1.45	10.1	8.56	0.254
RAJSSC Zhang et al. (2015)	0.57	4.23	1.75	13.87	9.05	0.242
RobStruck†	0.49	11.45	1.58	14.82	13.14	0.220
DSST Danelljan et al. (2014)	0.53	8.05	2.72	26.02	17.04	0.172
SAMF Li and Zhu (2014)	0.51	7.98	2.08	18.08	13.03	0.202
KCF Henriques et al. (2015)	0.47	12.83	2.43	21.85	17.34	0.171
NCC*	0.48	12.47	8.18	50.33	31.4	0.080

### 4.3 Surgical instrument tracking

PAWSS is a general tracking framework, we also want to evaluate its performance on both *ex vivo* and in vivo surgical instrument sequences. In the Endoscopic vision MICCAI2015 Challenge.,^4^ one of the sub-challenge focuses on comparing different vision-based methods for tracking conventional and articulated laparoscopic instruments in robotic surgery. The dataset has not released ground truth for test data. The official evaluation categorized conventional laparoscopic instrument test set according to the challenging factors including bleeding (C_blood_), smoke (C_smoke_), instrument occlusions (C_occlusion_), multiple instruments (C_multiple_) and surgical objects such as meshes and clips (C_objects_). And the robotic laparoscopic instrument dataset includes sequences with multiple instruments (C_multiple_). For evaluating the tracking performance, Euclidean distance of the centre point between the ground truth and the tracking result of training data is computed and compared separately for these challenging factors. We submitted our proposed method to the challenge, and obtained the performance comparison from the official report.

**Fig. 12:**
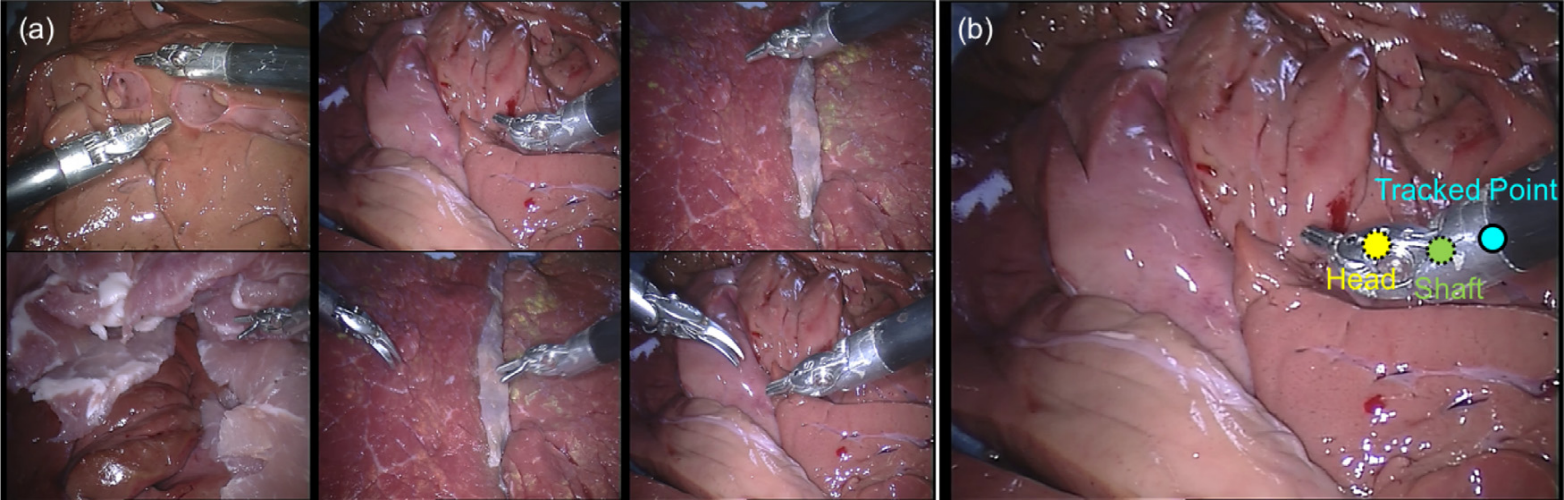
(a) Example frame from each sequence of *EndoVis* articulated surgical instrument dataset; (b) The original annotation includes the position of the tracked point, in our annotation, we relabeled the tracked point and also added new annotations for the Head and Shaft points.

***EndoVis*** **Articulated Robotic Laparoscopic instrument dataset**    The articulated instrument dataset is from ex vivo interventions, and the sequences are collected using the da Vinci^®^ (Intuitive Surgical Inc., CA) system with porcine tissue samples. Example frames from each sequence are shown in Fig. 12 (a). The dataset is divided into training and test data. Training data contains four 45 seconds surgery video sequences. For each instrument, the tracked point of the instrument is defined as the intersection between the instrument axis and the border between the shaft and the manipulator. The annotation includes pixel coordinates of the tracked point (Fig. 12 (b)). Test data is composed of 15 additional seconds video from each of the training sequence, and two additional new 60 s video sequences.

**Original annotation** We have summarized the frame number for each sequence and have shown the accuracy evaluation separately in the original annotation section of Table 6 and Fig. 14 Left. The accuracy is defined as the percentage of tracked frames within the error threshold. Distance (pixels) is averaged over correctly tracked frames. In Fig. 14, it shows accuracy under different threshold. In four train sequences, there are five instruments to be tracked. The average accuracy score for train data is 79.01% for 20 pixel threshold, with a distance error of 8.00 pixels. It is noted that the accuracy score (36.55% for 20 pixel threshold) for sequence 4 is relatively lower compared with the rest sequences. As we have summarized, the target is out of view several times in sequence 4, reaching 67 frames out of 1123 frames. Tracking-by-detection methods typically cannot handle out-of-view scenario without additional re-detection module. The underlying assumption is that the target is always in frame view, which means Whenever the target is out of frame, the tracker will gradually drift away. This explains the low accuracy of the performance, if the threshold is increased to 30 pixels, the performance has significantly improved, achieving 82.67% for accuracy.

**Table 6:** Accuracy of *EndoVis* articulated robotic surgical instrument training data for the tracked point.

	Seq 1L	Seq 1R	Seq 2	Seq 3	Seq 4	Whole
**Original annotation**						
In-view (IV) and Out-of-view (OV) Frame Number						
IV	1107	1107	1096	1118	1056	5484
OV	0	0	29	6	67	102
Total	1107	1107	1125	1124	1123	5586
Accuracy (Thres=20 px)						
Acc. (%)	85.00	92.86	90.60	88.10	36.55	79.01
Dist. (px)	7.42	7.07	7.41	9.64	9.26	8.00
Accuracy (Thres=30 px)						
Acc. (%)	99.37	96.93	96.35	95.80	82.67	94.33
Dist. (px)	9.76	7.80	8.36	10.71	18.07	10.67
**High quality annotation**						
In-view (IV) and Out-of-view (OV) frame number						
IV	1107	1107	1099	1105	1066	5484
OV	0	0	26	19	57	102
Total	1107	1107	1125	1124	1123	5586
Accuracy (Thres=20 px)						
Acc. (%)	100.0	99.73	98.91	98.28	95.78	98.56
Dist. (px)	4.89	9.87	3.29	4.31	11.13	6.65
Accuracy (Thres=30 px)						
Acc. (%)	100.0	100.0	99.36	99.46	99.72	99.71
Dist. (px)	4.89	9.90	3.38	4.56	11.57	6.83

**Fig. 13:**
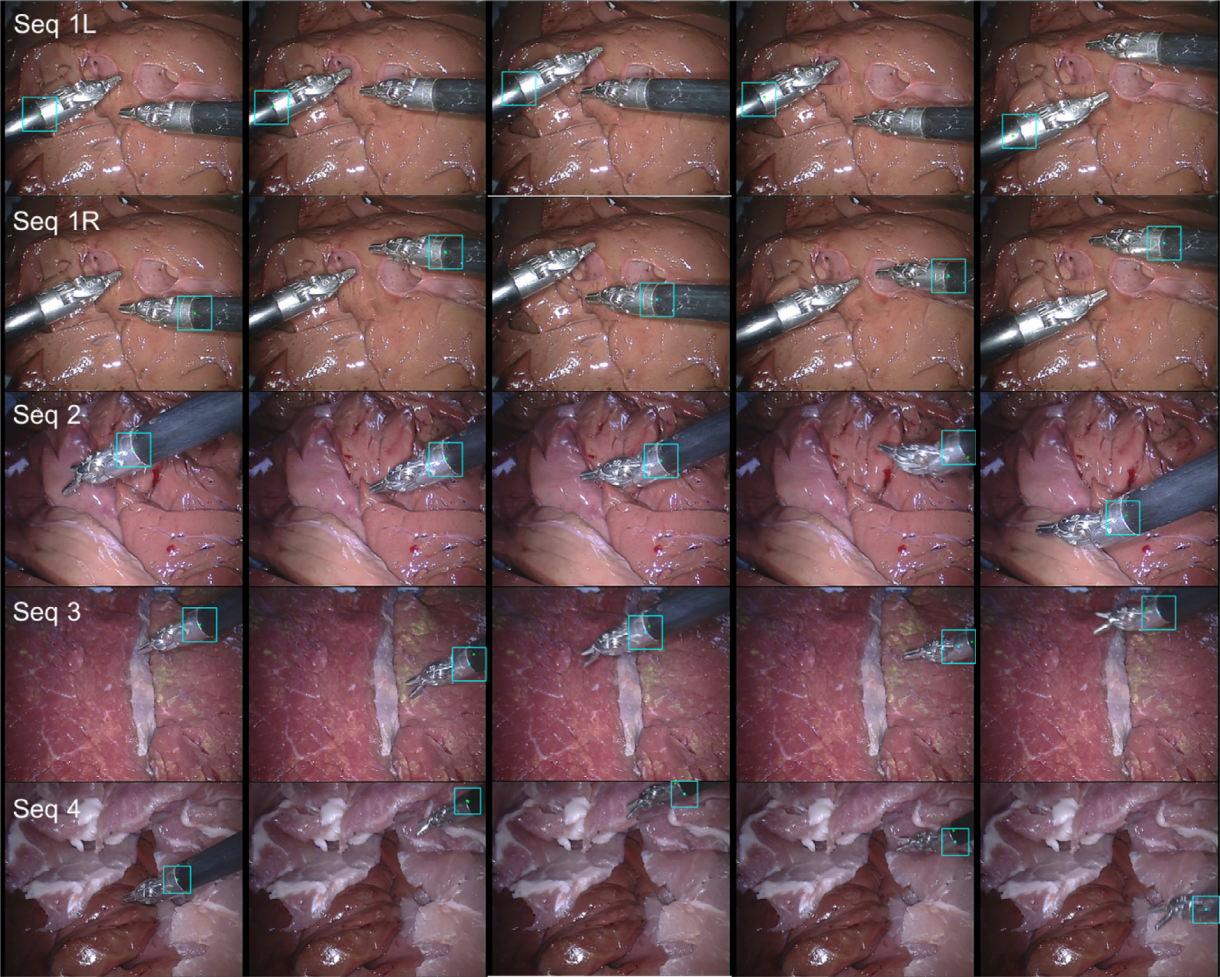
Result example frames from each sequence of the *EndoVis* articulated robotic surgical instrument dataset. The result bounding box and centre point is represented in cyan colour, and the ground truth centre point is represented in green colour. (For interpretation of the references to colour in this figure legend, the reader is referred to the web version of this article.)

We show some tracking result examples in Fig. 13. The tracked point and bounding box are shown in cyan colour, with the ground truth point shown in green colour. The first column is the first frame of each sequence. As we can see, the quality of the annotation is not consistent through the whole sequence. On certain frames, the annotation is drifted and is not labelled where it is supposed to be. This would certainly affect our performance evaluation result. It is also observed that whenever the instrument is close to the frame border, the tracker will stick to the border and not track the instrument well.

**Fig. 14:**
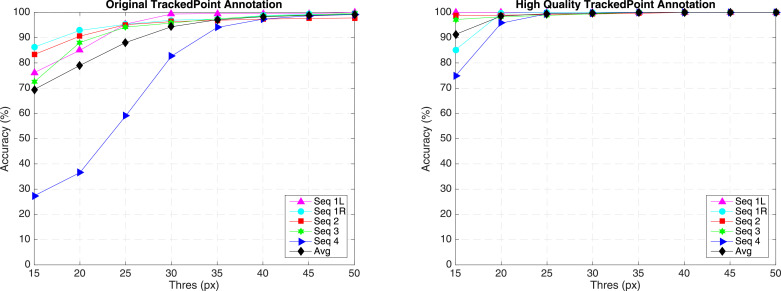
Tracking accuracy of *EndoVis* Articulated Robotic Surgical Instrument training data under different accuracy threshold with the original and high-quality annotations.

**High quality annotation** The original annotation is retrieved from the robotic system, which includes the location of the intersection point between the instrument axis and the border between plastic and metal on the shaft, normalized Shaft-to-Head axis vector and the clasper angle. Since the original annotation does not provide consistent ground truth, the accuracy result does not reflect true performance. We manually labelled the training data, and construct a high quality annotation. In this annotation, we labelled multiple joints of the instrument including the original tracked point, the Head and Shaft point. The original and our proposed annotations are demonstrated in Fig. 12 (b).

We also tracked and evaluated on the Head and Shaft points we defined in our high quality annotation in the high quality annotation section of Table 6 and Fig. 14 right. With new annotation, our average accuracy has increased to 98.56% for 20 pixel threshold, with distance error of 6.65 pixels.

**Fig. 15:**
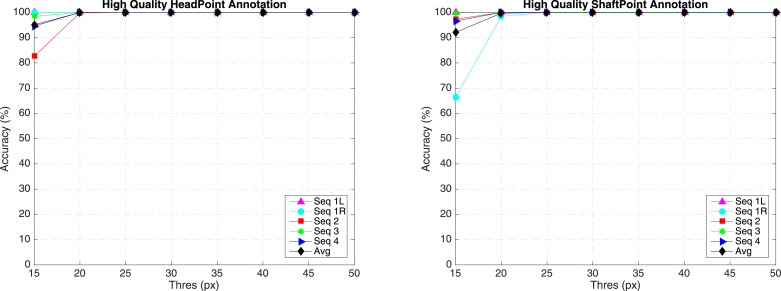
Accuracy of *EndoVis* Articulated Robotic Surgical Instrument training data under different accuracy threshold with high quality annotation.

The tracking accuracy evaluation results are displayed in Table 7 and Fig. 15. Our average accuracy has reached 99.96% and 99.68% for 20 pixels threshold, with distance error of 5.68 and 6.51 pixels, respectively.

**Table 7:** Accuracy of *EndoVis* articulated robotic surgical instrument train data for Head and Shaft points with high quality annotation.

	Seq 1L	Seq 1R	Seq 2	Seq 3	Seq 4	Whole
In-view (IV) and Out-of-view (OV) frame number						
IV	1107	1107	1125	1124	1123	5586
OV	0	0	0	0	0	0
Total	1107	1107	1125	1124	1123	5586
Head accuracy (Thres=20 px)						
Acc. (%)	100.0	100.0	99.82	100.0	100.0	99.96
Dist. (px)	3.06	4.10	10.32	4.52	6.33	5.68
Shaft accuracy (Thres=20 px)						
Acc. (%)	100.0	98.46	100	99.91	100	99.68
Dist. (px)	2.48	12.08	6.82	4.79	6.48	6.51

**Comparison performance** In Table 8, the distance error (pixel) was computed and compared separately for challenging factor multiple instrument (C_multiple_) with all the submitted methods KIT, UGA, MOD and our method PAWSS. From official report, PAWSS outperforms all the other methods with the lowest average distance error 29.66 pixels.

**Table 8:** Distance (pixel) comparison with all the submitted methods for the tracked Point of the robotic laparoscopic instrument test set. Multiple instrument challenging subset is evaluated separately.

	**C**_multiple_	Whole
KIT	113.91	106.60
UGA	40.73	34.94
MOD	45.12	40.16
PAWSS	**38.36**	**29.66**

**Fig. 16:**
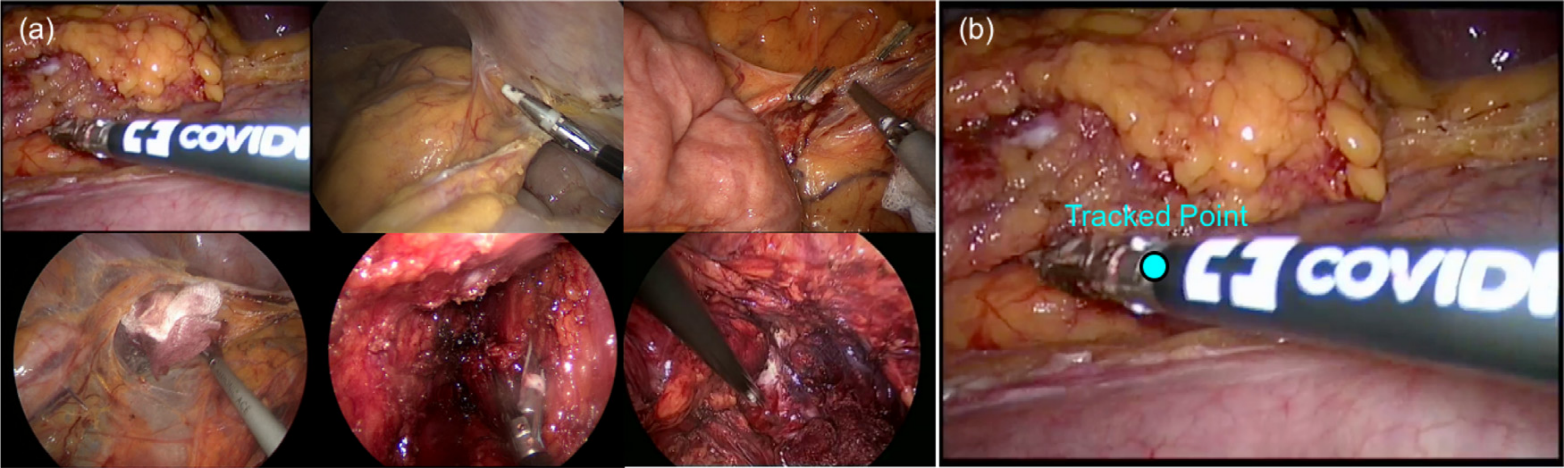
(a) Example frame from each sequence of *EndoVis* articulated surgical instrument training dataset; (b) The annotation includes the position of the tracked point.

**Fig. 17:**
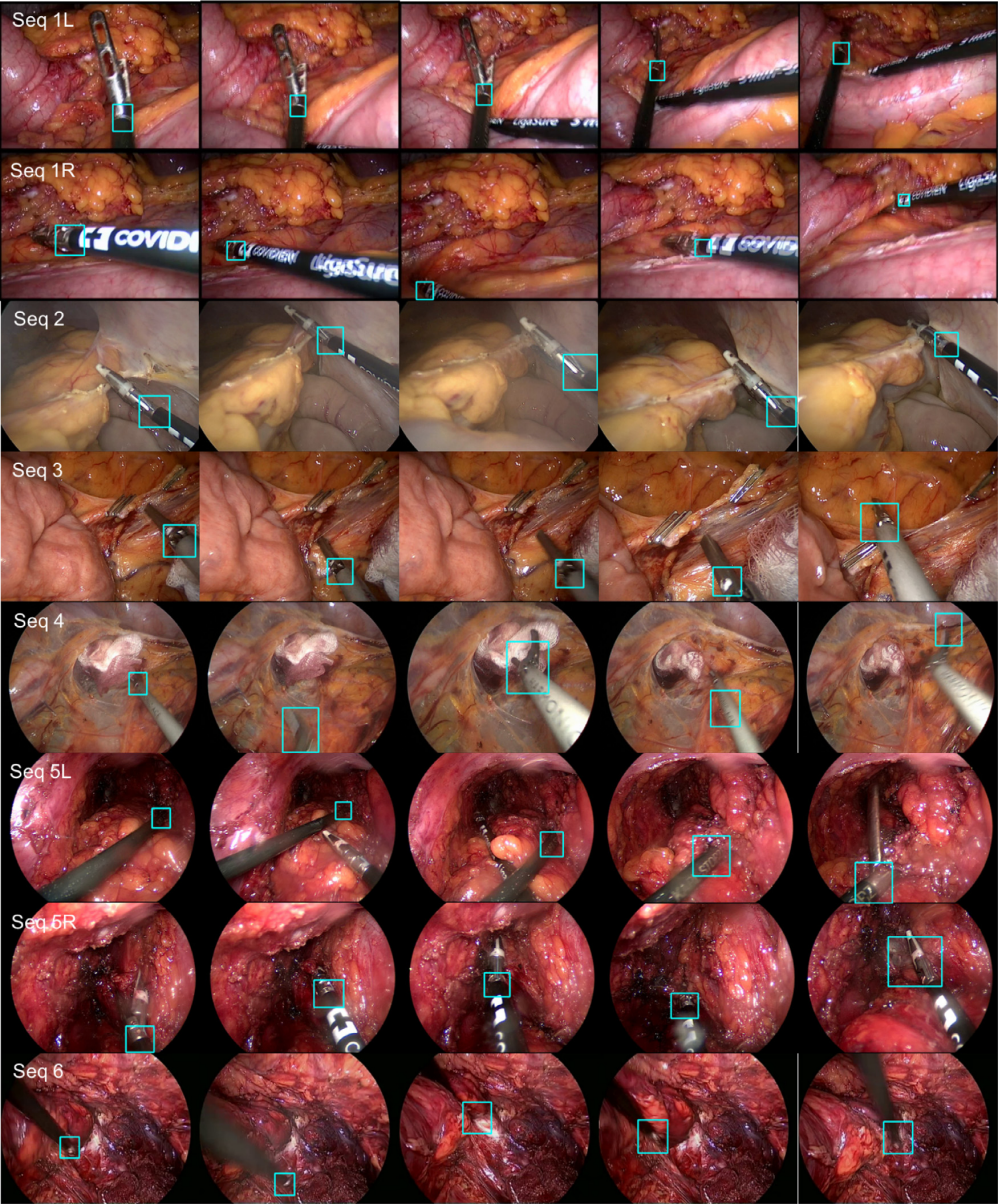
Result example frames from each test sequence of the *EndoVis* conventional surgical instrument dataset. The result bounding box is represented in cyan colour.

***EndoVis*** **Conventional Laparoscopic Instrument Dataset** The conventional instrument dataset contains six in vivo sequences, which are collected from complete laparoscopic colorectal interventions. Similar to the robotic instrument dataset, training data contains 45 s video sequences, and test data is made up of 15 additional seconds videos for each sequence and two new 60 s videos. Compared to *ex vivo* robotic instrument dataset, these sequences reflect complex challenges during surgery, including smoke, bleeding, blurry and various kinds of instruments. In Table 9, the distance error (pixel) was computed and compared separately for each challenging factor with all the submitted methods KIT, UGA and our method PAWSS. From the official report, PAWSS outperforms all the other methods in every challenging subset with the lowest average distance error 96.78 pixels. We show some tracking result examples in Fig. 17. The tracked point is shown in cyan colour, and the first column is the first frame of each sequence in test set. (Fig. 16)

**Table 9:** Distance (pixel) comparison with all the submitted methods for the tracked point of the conventional laparoscopic instrument test set. Various challenging subsets are evaluated separately.

	**C**_blood_	**C**_multiple_	**C**_objects_	**C**_occlusion_	**C**_smoke_	Whole
KIT	233.62	220.87	117.23	225.58	193.85	178.89
UGA	276.44	235.42	228.04	193.82	231.87	217.91
PAWSS	**181.59**	**110.85**	**68.29**	**87.11**	**96.31**	**96.78**

**Fig. 18:**
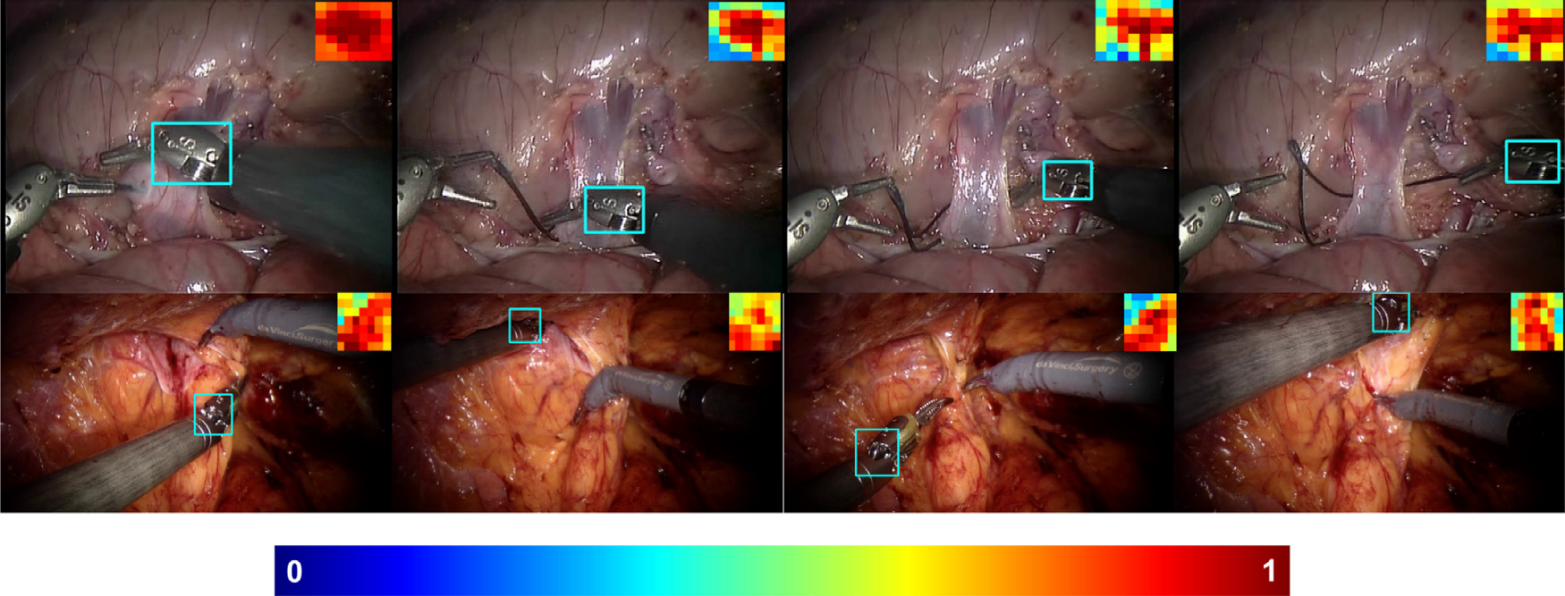
Instrument Tracking result with patch weight displayed in the top corner of the image.

**In vivo surgical instrument experiments** We also test on some other in vivo sequences and show the result in Fig. 18. As we can see, the tracker works well even under complex in vivo environment. The video is submitted to display the tracking results for the whole sequences.

## 5 Conclusions

In this paper, we propose a tracking-by-detection framework, called PAWSS, for online object tracking. It uses a colour-based segmentation model to suppress background information by assigning weights to the patch-wise descriptor. We incorporate scale estimation into the framework, allowing the tracker to handle both incremental and abrupt scale variations between frames. The learning component in our framework is based on Struck, but we would like to point out that theoretically our proposed method can also support other online learning techniques with effective background suppression and scale adaption.

The performance of our tracker is thoroughly evaluated on OTB, VOT2014 and VOT2015 datasets and compared with recent state-of-the-art trackers. Results demonstrate that PAWSS achieves the best performance in both PR and SR in OPE for OTB dataset. It outperforms Struck by 36.7% and 36.9% in PR/SR scores. Also, it provides a comparable PR score, and improves SR score by 4.8% over SOWP. On VOT2014 dataset, PAWSS has relatively lower accuracies but the lowest failure rate among the top trackers, we evaluated without re-initialization, and achieves the highest performance. Also on VOT2015 dataset, PAWSS is considered state-of-the-art and is among the top trackers.

For instrument tracking, we also qualitatively and quantitatively evaluated our tracker on public *EndoVis* robotic and conventional surgical instrument datasets, and in vivo surgical instrument sequences. We compared our result with the official GT for the Tracked Point on the robotic instrument dataset, and tracking accuracy reached 79.01% with 20 pixel threshold. As we have shown, the official annotation is not quality consistent, we manually created a high quality multi joint annotation for the dataset. We tested multiple joints (Tracked Point, Head and Shaft Point) on the dataset, and our performance accuracy increased over 98% for all the joints with 20 pixel threshold. From the official challenge report, Our method has shown the lowest tracking error for both robotic and conventional instrument datasets, and it also shown its excellent tracking ability with in vivo sequences dealing with complicated surgical environment. Our framework is designed for general single object tracking. It does not require prior information about the target or any offline training to achieve robust and real-time performance. We would also like to discuss the limitations of our framework. First, if the target disappears and reappears from the scene, the framework does not recover. Second, the target position is represented by rectangle bounding box. Even with the assistance of the segmentation model to distinguish foreground and background, the assumption is that the target occupies most area of the bounding box. If the target only occupies small fraction, the classifier would be polluted and misled by background information and can easily cause tracking failure. In the future, we would like to focus on re-detection module and semantic foreground segmentation.

## Declarations of interest

None

## Appendix A Supplementary materials

Supplementary Data S1 Supplementary Raw Research Data. This is open data under the CC BY license http://creativecommons.org/licenses/by/4.0/ Supplementary Data S1

Supplementary Data S2 Supplementary Raw Research Data. This is open data under the CC BY license http://creativecommons.org/licenses/by/4.0/ Supplementary Data S2

## Footnotes

1 https://github.com/surgical-vision/PAWSS.

2 http://www.votchallenge.net/.

3 This is an improved version of the original tracker.

4 https://endovissub-instrument.grand-challenge.org/.
