# Supplementary figures and images for: Patch-based adaptive weighting with segmentation and scale (PAWSS) for visual tracking in surgical video
Source: Med Image Anal. 2019 Oct;57:120–35. doi: 10.1016/j.media.2019.07.002 (PMC6988132; doi:10.1016/j.media.2019.07.002)

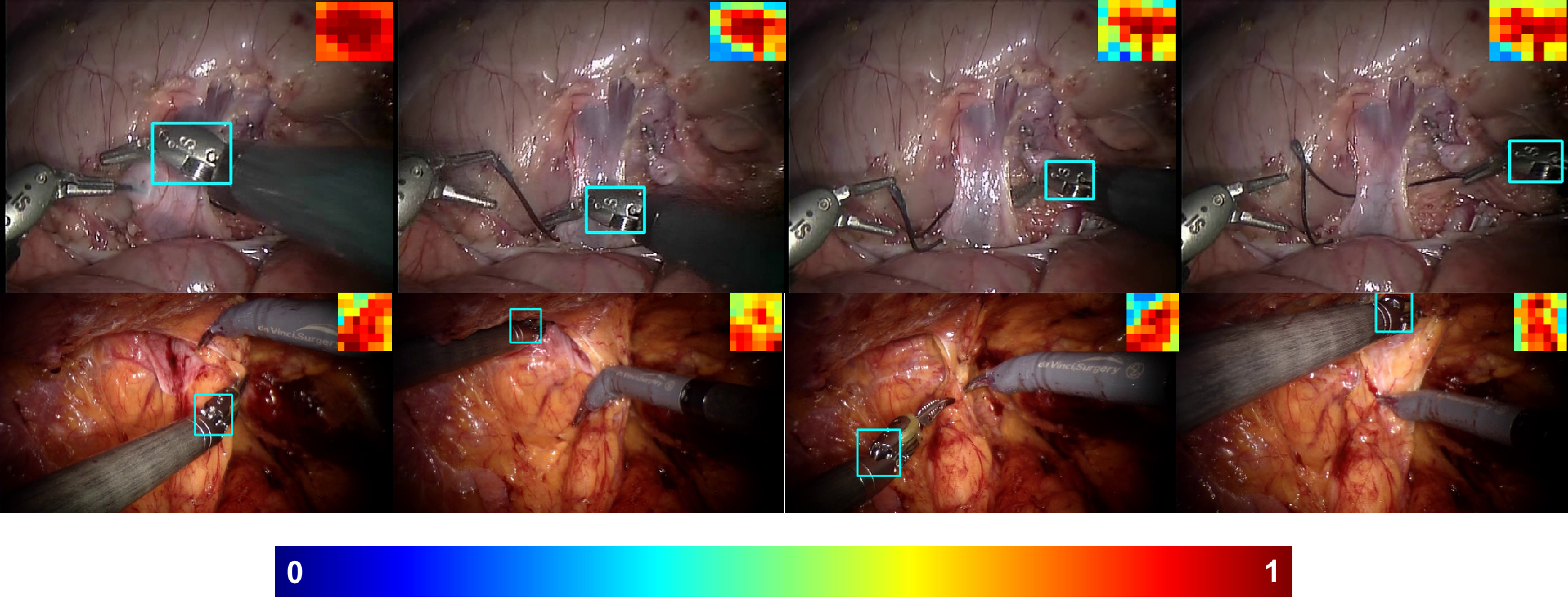

Supplement: Supplementary Data S2 — Supplementary Raw Research Data. This is open data under the CC BY license http://creativecommons.org/licenses/by/4.0/ [file mmc2.jpg]
